# Caring for carers; preventing the mental health issues of future health care professionals

**DOI:** 10.1192/j.eurpsy.2026.11856

**Published:** 2026-07-31

**Authors:** A. Arshad, A. Sheraz, R. Kokab, G. S. Ghouri

**Affiliations:** 1 Allama Iqbal Medical College; 2 Services Institute of Medical Sciences Lahore; 3 King Edward Medical University, Lahore, Pakistan

## Abstract

**Introduction:**

Medical students face a lot of academic pressures and psychological distress, exacerbated by sleep deprivation and exposure to human suffering. In South Asia, stigma associated with mental health along with limited awareness and scarce resources further increase the challenges. Most Pakistani educational institutes do not have mental health counsellors. In this study we aimed to take a snapshot of depression in medical students of two colleges to understand the burden and inform strategies for support.

**Objectives:**

To determine the prevalence of depression among medical students in two medical colleges and to assess their access to mental health facilities.

**Methods:**

A total of 376 medical students from AIMC and RMU participated in this mental health survey; 267 from Allama Iqbal Medical College (AIMC) and 109 from Rawalpindi Medical University (RMU). Mental health of these students was assessed using Patient Health Questionnaire (PHQ) scale based on their responses, and diagnostic categorization was based on the ICD-11 criteria. Responses were gained from across all academic years, with a greater female to male ratio of participants of 2:1.

Notably, 82% of participants believed that perceived stigma attached with mental health in Pakistan is a significant issue.

**Results:**

Out of 376 participants, 56 students (14%) met criteria for severe depression, 98 (26%) for moderate depression, and 46 (12%) for mild depression, and 176 (47%) students did not meet the criteria for depression.

Depression was significantly associated with advances years of medical studies (p = 0.04), while institutional differences were not significant (p = 0.269). Female students were more likely to experience depression as compared to males (p= 0.02).

**Conclusions:**

Our findings highlight a substantial prevalence of depression among Pakistani medical students. These statistics emphasize the immediate need for mental health facilities to be setup in universities and medical colleges under professional supervision. As future healthcare providers, medical students must be supported to ensure their well being, Its time to prioritize ‘caring for carers’.

**Disclosure of Interest:**

None Declared

